# Food deprivation exposes sex‐specific trade‐offs between stress tolerance and life span in the copepod *Tigriopus californicus*

**DOI:** 10.1002/ece3.8822

**Published:** 2022-04-12

**Authors:** Ning Li, Ben A. Flanagan, Suzanne Edmands

**Affiliations:** ^1^ 5116 Department of Biological Sciences University of Southern California Los Angeles California USA

**Keywords:** aging, food limitation, interpopulation hybrid, longevity

## Abstract

Long life is standardly assumed to be associated with high stress tolerance. Previous work shows that the copepod *Tigriopus californicus* breaks this rule, with longer life span under benign conditions found in males, the sex with lower stress tolerance. Here, we extended this previous work, raising animals from the same families in food‐replete conditions until adulthood and then transferring them to food‐limited conditions until all animals perished. As in previous work, survivorship under food‐replete conditions favored males. However, under food deprivation life span strongly favored females in all crosses. Compared to benign conditions, average life span under nutritional stress was reduced by 47% in males but only 32% in females. Further, the sex‐specific mitonuclear effects previously found under benign conditions were erased under food limited conditions. Results thus demonstrate that sex‐specific life span, including mitonuclear interactions, are highly dependent on nutritional environment.

## Graphical abstract

The copepod *Tigriopus californicus* defies the expected positive relationship between stress tolerance and life span, with stress‐sensitive males generally living longer than females in benign conditions. Exposure to food limitation beginning at adulthood restores the expected longer life of stress‐tolerant females in all four crosses tested.

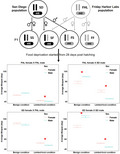

## 1 INTRODUCTION

A long‐standing dogma in biology is that the ability to withstand stress is associated with longer life (Kirkwood & Austad, 2000). This is supported by overlap in the genetic bases for these two traits, including the roles of molecular chaperones, antioxidants, and genes involved in repair of oxidative damage (Landis et al., 2004; Vermeulen & Loeschcke, 2007). More direct evidence comes from artificial selection experiments, in which selection for longer life span increases resistance to stressors such as starvation, desiccation, ethanol, and high temperature (Scannapieco et al., 2009; Service et al., 1985), and selection for increased stress resistance (desiccation, starvation, and high temperature) also increases longevity (Lind et al., 2017; Pijpe et al., 2008; Rose et al., 1992).

The copepod *Tigriopus californicus* provides a counter example to the expected positive relationship between stress tolerance and life span. Females are more tolerant than males to a range of stressors (Foley et al., 2019; Kelly et al., 2012; Willett, 2010) and also exhibit a substantially muted transcriptomic response to oxidative stress (Li et al., 2019, 2020). While females might be expected to also have longer lives, the first large‐scale study of longevity in this species (Flanagan et al., 2021) showed that life span under benign conditions is either equivalent between sexes or longer in males. Further, comparisons of two parental lines and their reciprocal F1 hybrids revealed sex‐specific mitonuclear effects on longevity. Such mitonuclear interactions are perhaps unsurprising, given that the two parental lines have widely divergent mitochondrial haplotypes (20.6%; Barreto et al., 2018) and that mitochondrial function is known to involve interactions with over 1000 proteins encoded by the nuclear genome (Bar‐Yaacov et al., 2012). Because *T. californicus* does not have sex chromosomes (Alexander et al., 2015; Voordouw & Anholt, 2002), it offers a simpler system for testing sexually dimorphic mitochondrial effects, as asymmetric inheritance of mitochondria is not confounded with asymmetric inheritance of sex chromosomes.

Here, we extend previous work (Flanagan et al., 2021) to assess sex‐specific effects of nutritional stress. By using the same parental and reciprocal crosses, we test how mitochondrial effects are altered by environment. Given that mitochondria are the location where dietary nutrients are converted to ATP, mitochondrial effects might be expected to highly dependent on nutritional environment. In this study, we use an additional clutch of offspring from the same families assayed in Flanagan et al. (2021), raising animals under benign conditions until adulthood, and then transferring them to culture medium without food. In this way, we test how nutritional stress impacts sex differences in longevity, including the role of mitochondrial effects.

## 2 MATERIALS AND METHODS

Populations were collected from San Diego, CA (S: 32.75°N, 117.25°W), and Friday Harbor Laboratories, WA (F: 48.55°N, 123.01°W). They were kept in a 20°C incubator with a 12h:12h light:dark cycle. Isofemale lines from each population were established from single ovigerous females and inbred for a minimum of ten generations before experiments began. Lines were maintained in petri dishes (diameter × height = 100 mm × 15 mm) in natural filtered seawater (37 µm) supplemented with a mixture of powdered Spirulina (Nutrex Hawaii) and ground Tetramin flakes (Tetra) at a concentration of 0.1 g of each food per L.

**FIGURE 1:**
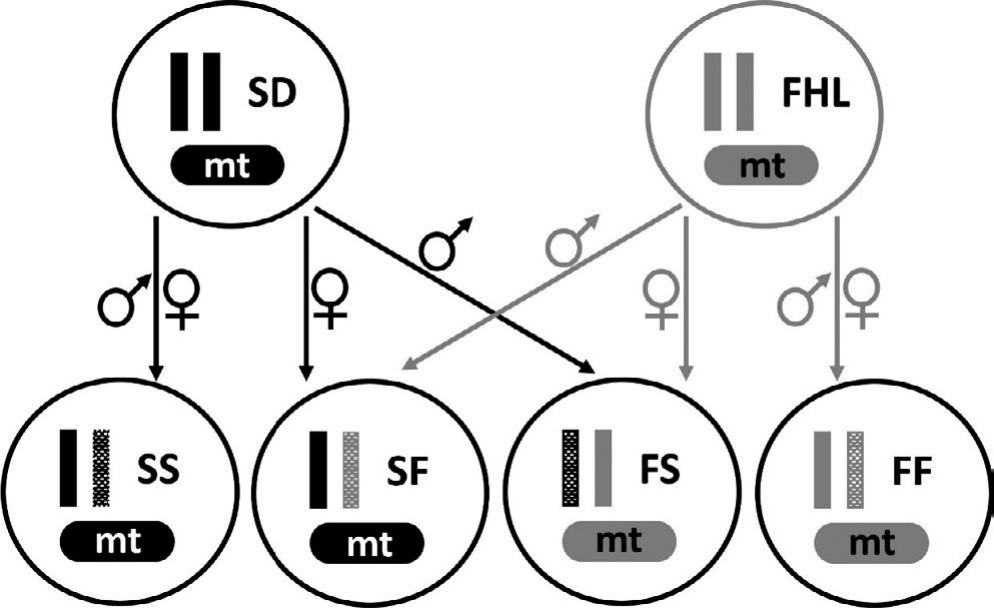
Crossing design using inbred lines from San Diego, CA (SD/S), and Friday Harbor Laboratories, WA (FHL/F). SD nuclear and mitochondrial (mt) genomes are indicated in black, and FHL nuclear and mitochondrial genomes are indicated in gray. Solid bars indicate nuclear alleles contributed by the mother, and dotted bars indicate nuclear alleles contributed by the father. F1 generation cohorts were named as dam × sire

*Tigriopus californicus* mature males clasp virgin females using their antennae and remain clasped until the females become reproductively mature (Burton, 1985; Egloff, 1966; Vittor, 1971). Therefore, virgin females can be obtained by teasing apart the clasped pair on a moist filter paper under a dissecting microscope using fine probes. This technique has been tested to be satisfactory with few individuals injured and no impaired brood production during the handling procedure (Burton, 1985; Vittor, 1971). As described in Figure 1, within‐population crosses (FF cross: F female mated with F male, and SS cross: S female mated with S male) and reciprocal, between‐population crosses (FS cross: F female mated with S male, and SF cross: S female mated with F male) were set up by combining virgin females with mature males from the designated populations. Only one female and one male were allowed in one petri dish, and they usually form a pair within one day. The culture medium was the same as the original population cultures. This experiment used the fourth clutch of the crosses generated in Flanagan et al., 2021 for direct comparisons with the survival and longevity under benign conditions. Briefly, males were removed to avoid further harassment after the females were released from the pair. New crosses were set up to replace the ones whose individuals died or whose females were not successfully fertilized. Petri dishes were checked every day until the fertilized egg sacs hatched. The offspring from the first three egg clutches were collected and assayed under benign conditions (Flanagan et al., 2021), while the offspring from the fourth clutch were then counted and transferred to a new petri dish for this study. The estimated effect of clutch number on survival was minimal during the previous experiment for the first three clutches under benign conditions (coxph, z = −1.94, *p* = .052) (Flanagan et al., 2021). Further, prior work in *T. californicus* failed to detect an effect of clutch number on the proportion of surviving individuals fourteen days after hatching, although later clutches had fewer offspring and smaller body size (Powers et al., 2020).

The larvae were fed and rehydrated once every week until 28 days post‐hatching, at which time the females and males could be distinguished from the structure of antennae (Egloff, 1966). The two sexes were counted and if animals had formed pairs, they were separated using fine probes as described above. At day 28 post‐hatching, the food limitation treatment was initiated by transferring males and females into separate petri dishes with filtered seawater only. We characterize this as a food limitation treatment rather than a starvation treatment since the coarsely filtered seawater (37 µm) is expected to contain microbes and also support some algal growth. To measure survivorship and maximum life span under food limitation, animals were counted every day beginning on day 28 and dead individuals were removed until all individuals died.

In total, 15 FF families, 8 FS families, 12 SF families, and 13 SS families were used for this study. Survival analysis was conducted by Kaplan–Meier analysis using the survival package v3.1‐12 in R version 3.5.1. Survivorship was also fit to a cox‐proportional hazard model with mixed effects (Therneau, 2020) using the coxme package v2.2‐16 in R version 3.5.1. Within crosses and for all crosses combined, sex was modelled as the fixed effect and family as the random effect to estimate the hazard ratio, indicating the ratio of male death hazard relative to females.

## 3 RESULTS AND DISCUSSION

**FIGURE 2:**
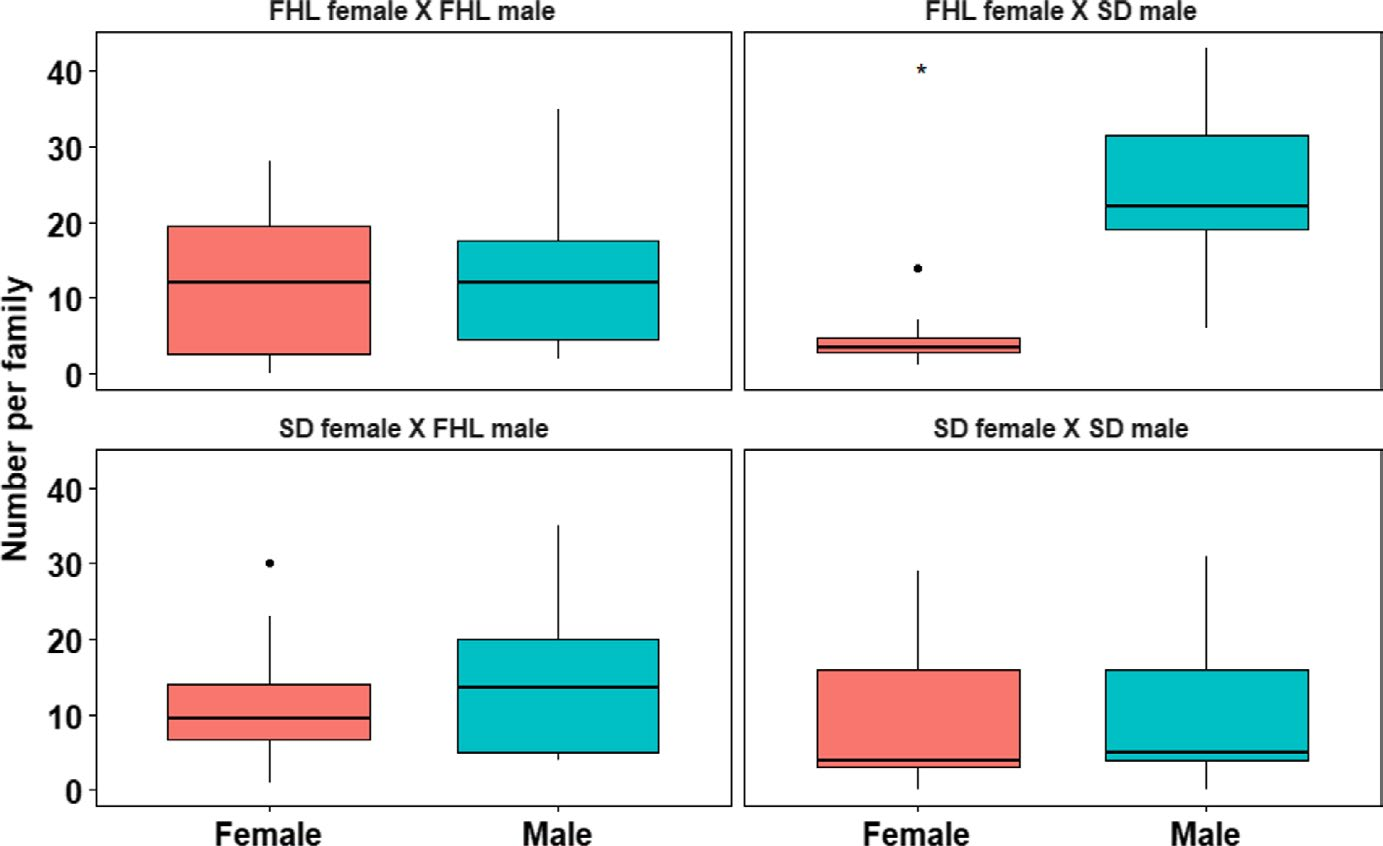
Sex ratio for each cross independently represented as the number of each sex per family (female—red; male—blue) under limited food conditions. Asterisks indicate significance level by paired samples Wilcoxon test (*p < .05)

In this study, we used an additional clutch of offspring from the same families used in Flanagan et al. (2021), raising them in the same benign conditions until day 28 posthatching. At this time point, sex ratios in Flanagan et al. (2021) and the current study were similar (Figure 2). In both cases, ratios in the four crosses were either equivalent or male‐biased.

**FIGURE 3:**
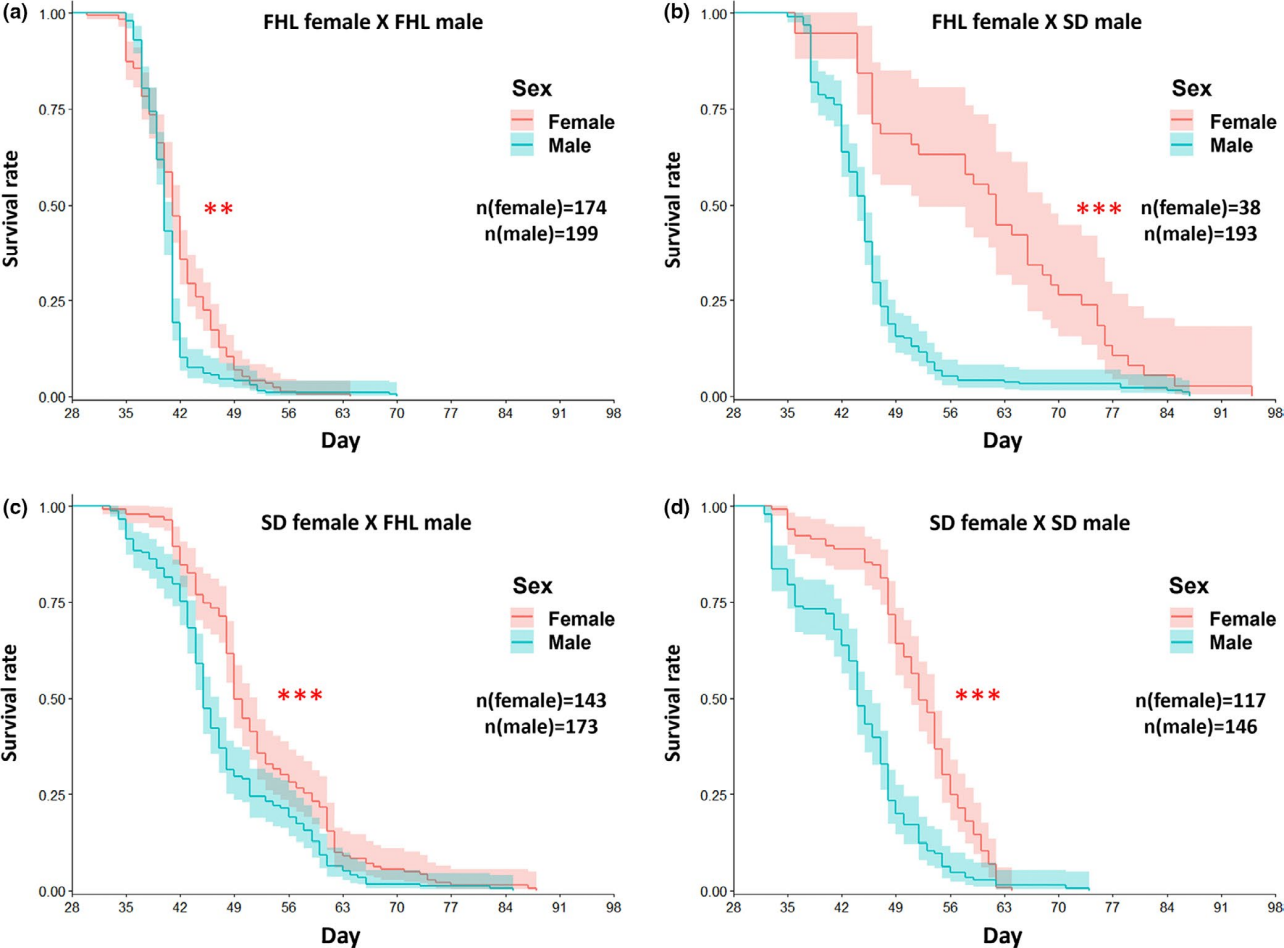
Kaplan–Meier curve with 95% confidence bands displaying the estimated survival probability for crosses FF (a), FS (b), SF (c), and SS (d) under limited food conditions. The number of female and male individuals used in this study was listed in each panel. Asterisks indicate significance level (**p‐value <.01; *** *p*‐value <.001) between sexes by a cox‐proportional hazard model with mixed effects (coxme)

After day 28, copepods were sexed and either maintained in benign conditions (Flanagan et al., 2021) or transferred to food‐limited conditions (current study) until all animals perished. Here, results of the two studies were dramatically different. Under benign conditions (Table 1), combined results show increased male longevity, with males having longer average life span, longer average maximum life span, and higher overall survival (coxme, χ^2^ = 14.23, *p* = 1.62 e‐4). Sex‐specific life span differs between the four crosses, including higher overall survival for males in one F1 cross (FS) and sex‐equivalent survival in the other F1 cross (SF). Because these reciprocal F1 have different mitochondrial haplotypes on a 50:50 nuclear background, with nuclear contributions from different parental crosses, this suggests sex‐specific mitochondrial effects. Results under limited food conditions (Table 1 and Figure 3) are strikingly different, revealing increased female longevity for nearly all metrics. Combined results showed females having longer average life span, longer average maximum life span and higher overall survival (coxme, χ^2^ = 287.60, *p* < 2.20 e‐16). Overall survival also favored females in each of the four crosses, suggesting that higher female tolerance to nutritional stress overrides the sex‐specific mitonuclear interactions inferred under benign conditions.

**TABLE 1:** Life span comparisons between benign conditions and limited food conditions in each sex within each cross

Cross	Sex	Benign conditions^a^			Limited food conditions		
		Average life span	Maximum life span^b^	Overall survival^c^	Average life span	Maximum life span^b^	Overall survival^c^
FF	Female	52.7 ± 0.6	80.5 ± 6.4	No difference	41.8 ± 0.4	48.6 ± 2.1	Female > Male*
	Male	56.9 ± 0.7	90.7 ± 7.7		40.4 ± 0.3	44.3 ± 2.0	
FS	Female	78.0 ± 2.5	107.8 ± 9.6	Male > Female*	60.8 ± 2.3	73.0 ± 4.3	Female > Male*
	Male	89.6 ± 0.8	135.2 ± 8.3		45.7 ± 0.6	60.9 ± 4.9	
SF	Female	91.2 ± 1.3	131.0 ± 9.2	No difference	52.0 ± 0.8	62.4 ± 3.4	Female > Male*
	Male	88.3 ± 1.2	113.4 ± 7.4		47.5 ± 0.7	57.6 ± 4.1	
SS	Female	85.2 ± 2.2	111.4 ± 10.6	Male > Female*	51.7 ± 0.7	53.6 ± 2.8	Female > Male*
	Male	104.7 ± 1.9	140.9 ± 11.4		44.2 ± 0.7	51.1 ± 3.1	
Total	Female	71.5 ± 0.8	107.5 ± 5.0	Male > Female*	48.9 ± 0.5	57.8 ± 2.0	Female > Male*
	Male	84.0 ± 0.6	120.6 ± 4.8		44.3 ± 0.3	52.2 ± 1.9	

Note

Results are coded in red if higher in females and blue if higher in males. Data are shown as mean ± SEM. *p‐value <.01.

Abbreviations: FF, F female mated with F male; FS, F female mated with S male; SF, S female mated with F male; SS, S female mated with S male.

^a^ Data for benign conditions are from Flanagan et al., 2021.

^b^ Maximum life span is a family‐based calculation and estimated from all families within each cross.

^c^ Overall survival is based on analyses from a cox‐proportional hazard model with mixed effects.

Importantly, the food deprivation treatment (no added food beginning at adulthood) was detrimental to both sexes, reducing average life span by 31.6% in females and 47.3% in males. In contrast, other studies found that less drastic food limitation commonly extends life span. Moderate diet restriction, typically 10%–60%, has been found to increase longevity across a remarkably diverse range of organisms including yeast (Gouspillou & Hepple, 2013), nematodes (Gouspillou & Hepple, 2013), fruitflies (Krittika & Yadav, 2019), rodents (Kane et al., 2018), fish (Terzibasi et al., 2009), non‐human primates (Colman et al., 2009), *Daphnia* (Hearn et al., 2019), and copepods (Saiz et al., 2015), suggesting common underlying mechanisms. Such studies typically find that moderate diet restriction is more beneficial in females than males (Aw et al., 2017; Freire et al., 2020; Ingram & de Cabo, 2017; Magwere et al., 2004), paralleling our finding that food deprivation is less detrimental in females.

Higher female tolerance of food deprivation is a common pattern in arthropods in general (Gerofotis et al., 2019; Knapp, 2016; Matzkin et al., 2009) and copepods in particular (Finiguerra et al., 2013; Holm et al., 2018). A frequent explanation for the pattern is higher body size in females, a pattern found in many arthropods (e.g., Holm et al., 2018; Gerofotis et al., 2019) including *T. californicus* (Edmands & Harrison, 2003). Another contributing factor may be higher lipid reserves found in some females (e.g., Holm et al., 2018; Gerofotis et al., 2019), although sex differences in lipid content are not known for *Tigriopus*. A third contributing factor commonly cited for copepods is that males typically expend more energy on searching for mates (Finiguerra et al., 2013; Holm et al., 2018). This is likely the case in *T. californicus*, where males mate repeatedly while females mate only once (Burton, 1985). Higher female tolerance of food deprivation is so common in copepods that female‐skewed ratios can be used as an indicator of food scarcity in the wild (Finiguerra et al., 2013). Female bias is less detrimental to population viability than male‐bias (Edmands, 2021; Wedekind, 2002), but the extreme female bias found in some natural copepod populations can cause sperm limitation (Kiørboe, 2007).

In summary, under benign conditions *T. californicus* defies the expected positive relationship between stress tolerance and life span, with stress‐sensitive males generally living longer than females. Exposure to food limitation beginning at adulthood restores the expected longer life of females. This is true for all crosses, thus overriding the sex‐specific mitochondrial effects found under benign conditions. Importantly, food limitation may be the more common state for copepods in natural conditions. Results are consistent with work on *Drosophila* (Camus et al., 2012; Nagarajan‐Radha et al., 2019) in which sex‐specific life span, including effects of mitochondrial haplotype, are dependent on nutritional environment.

## CONFLICT OF INTEREST

All authors declare no competing interests.

## AUTHOR CONTRIBUTIONS

**Ning Li:** Conceptualization (lead); Formal analysis (lead); Methodology (lead); Supervision (equal); Visualization (lead); Writing – original draft (lead); Writing – review & editing (equal). **Ben A. Flanagan:** Data curation (equal); Formal analysis (equal); Methodology (equal); Resources (lead); Writing – review & editing (equal). **Suzanne Edmands:** Conceptualization (equal); Funding acquisition (lead); Methodology (equal); Project administration (lead); Supervision (equal); Writing – original draft (lead); Writing – review & editing (equal).

## Data Availability

All the data and scripts are available at Dryad (https://doi.org/10.5061/dryad.h44j0zpn1).
